# Dynamic blood dose estimates in radiotherapy and correlations with adverse clinical outcomes in brain, head‐and‐neck, and lung cancer patients

**DOI:** 10.1002/acm2.70341

**Published:** 2025-12-29

**Authors:** Sebastian Tattenberg, Jungwook Shin, Cornelia Hoehr, Xuanfeng Ding, Rohan L. Deraniyagala, Wonmo Sung

**Affiliations:** ^1^ School of Natural Sciences Laurentian University Sudbury Ontario Canada; ^2^ Life Sciences Division TRIUMF Vancouver British Columbia Canada; ^3^ Division of Cancer Epidemiology and Genetics National Cancer Institute National Institutes of Health Rockville Maryland USA; ^4^ Department of Radiation Oncology Beaumont Health Royal Oak Michigan USA; ^5^ Department of Biomedical Engineering College of Medicine The Catholic University of Korea Seoul South Korea; ^6^ Department of Medical Sciences Graduate School of The Catholic University of Korea Seoul South Korea

**Keywords:** blood dose, dose‐response relationship, radiation‐induced lymphopenia, radiotherapy

## Abstract

**Background:**

In cancer radiotherapy, radiation‐induced lymphopenia (RIL) has been reported to be correlated with adverse clinical outcomes such as reduced locoregional control (LRC), distant‐metastasis‐free survival (DMFS), and overall survival (OS) in various treatment sites. Frameworks to simulate the radiation dose to circulating blood have been developed in response, and simulated blood dose values have been reported to be correlated with severe RIL and/or adverse clinical outcomes. However, validations with different patient datasets or expansions to additional treatment sites, as well as the identification of particularly relevant blood dose metrics and blood compartments to allow for their inclusion during radiotherapy treatment planning, remain lacking.

**Purpose:**

This study aims to investigate a potential correlation between simulated blood dose values and adverse clinical outcomes in 215 patients with head‐and‐neck squamous cell carcinoma (HNSCC), 180 patients with glioblastoma (GBM), and 490 patients with non‐small‐cell lung cancer (NSCLC), and to identify particularly relevant blood dose metrics and blood compartments to allow for their inclusion during radiotherapy treatment planning and thereby enable the optimization of the estimated dose delivered to circulating blood.

**Methods:**

For all 885 patients, TotalSegmentator was used to automatically delineate additional organs‐at‐risk (OARs), blood vessels, and tissues which were not already manually delineated for radiotherapy treatment planning. Subsequently, the dynamic HEDOS model, which considers temporal aspects such as blood flow dynamics and treatment delivery time, was used to simulate the radiation dose delivered to circulate blood during radiotherapy. Static blood dose models consisting of the mean dose to the union of all HEDOS blood compartments (*D*_static,HEDOS_) and the integral body dose (*D*_static,body_) were also investigated to verify whether a simplified blood dose model equally exhibited any correlations with adverse clinical outcomes.

**Results:**

During multivariable Cox regression analysis, the blood dose estimates from the dynamic blood dose model exhibited a statistically significant (*p* < 0.05) correlation with DMFS and OS in the HNSCC and NSCLC datasets as well as with LRC in the HNSCC dataset. *D*_static,body_ and *D*_static,HEDOS_ only exhibited a statistically significant correlation with OS in the GBM and NSCLC datasets. Within a dataset, different dynamic blood dose metrics generally consistently exhibited correlations with the same clinical outcomes. Large arteries and veins were found to be a particularly relevant blood compartment within the HNSCC dataset, while the dose to the healthy portion of the brain and the dose to the heart and lungs were found to exhibit particularly strong correlations with dynamic blood dose estimates in the GBM and NSCLC datasets, respectively.

**Conclusions:**

The dynamic blood dose model exhibited a statistically significant correlation with adverse clinical outcomes in five out of seven cases, compared to just two cases for the static blood dose models. Consideration of temporal aspects such as blood flow dynamics and treatment delivery time was therefore essential to some of the observed correlations. For each treatment site, particularly relevant blood compartments were identified, allowing for their inclusion during radiotherapy treatment planning as part of future studies which aim to reduce the estimated dose to circulating blood.

## 1 INTRODUCTION

Radiation therapy is one of the most important treatment modalities for cancer, but radiation‐induced lymphocyte depletion, which has been reported to occur in 40%–70% of patients treated with fractionated external radiotherapy, is now receiving considerable attention.^1^ This is because radiation‐induced lymphopenia (RIL) has been reported to exhibit a statistically significant correlation with adverse clinical outcomes, including poorer locoregional control (LRC), distant‐metastasis‐free survival (DMFS), and overall survival (OS), in a variety of treatment sites.^2^, ^3^, ^4^, ^5^, ^6^, ^7^, ^8^, ^9^, ^10^, ^11^, ^12^ Treatment‐associated lymphopenia may result from direct radiation‐induced lymphocyte killing as well as the lymphotoxic effects of concurrent chemotherapy and steroids.^2^, ^13^, ^14^, ^15^ In addition to the direct effects on irradiated lymphocytes, indirect effects of radiation on lymphoid organs such as the spleen as well as on unirradiated bone marrow have also been reported.^16^, ^17^

Various models to estimate the radiation dose to circulating blood or lymphocytes during radiotherapy delivery have been developed over the years.^13^, ^18^, ^19^, ^20^, ^21^ Such models differ with respect to aspects such as their spatial and temporal resolution, but considerable improvements have been made recently, including the expansion to patient‐specific vasculature information and the development of an open‐source framework allowing for more widespread incorporation into radiotherapy research.^22^, ^23^, ^24^

However, to the best of our knowledge, studies which systematically investigate the correlation between model‐based blood or lymphocyte dose estimates and severe RIL or adverse clinical outcomes over multiple treatment sites remain outstanding. Estimates from the effective dose to immune cells (EDIC) approach have shown a statistically significant correlation with RIL in breast cancer patients, and additionally with adverse clinical outcomes in patients with esophageal cancer.^21^, ^25^ Blood dose estimates from the open‐source hematological dose (HEDOS) framework have likewise been reported to be correlated with severe RIL in hepatocellular carcinoma patients as well as with poorer clinical outcomes in patients with head‐and‐neck cancer.^26^, ^27^ However, a validation with an independent patient dataset for the same treatment site or an expansion to further treatment sites, as well as the identification of potentially relevant blood dose metrics and blood compartments which can be included during the radiotherapy treatment plan optimization with the aim of reducing the estimated dose to the circulating blood, are lacking thus far.

This study aims to build upon a previous work, which reported statistically significant correlations between HEDOS blood dose estimates and adverse clinical outcomes in a dataset consisting of 298 patients with head‐and‐neck cancer, and expand the developed methodology to a further 885 patients with three different treatment sites.^27^ This includes a further 215 head‐and‐neck cancer patients with squamous cell carcinoma (HNSCC), 180 patients with glioblastoma (GBM), and 490 patients with non‐small cell lung cancer (NSCLC). A comparison with three different static blood dose model approaches will also be performed, and particularly relevant dynamic blood dose metrics and blood compartments will be identified. By doing so, this work aims to further the community's understanding of the clinical utility of dynamic blood dose models and identify relevant blood compartments and blood dose metrics which can then be included during clinical radiotherapy treatment planning.^28^

## 2 METHODS

The workflow utilized for this study is in agreement with the approach utilized by a prior work.^27^ First, patient computed tomography (CT) scans were used for the automatic delineation of additional organs, blood vessels, and tissues which had not already been manually delineated for radiotherapy treatment planning, with the aim of allowing for the creation of a more realistic blood dose model. Subsequently, simulations of the radiation dose delivered to circulating blood were performed for all patients, based on the patient's CT scan, delineated structures, and their clinical external photon therapy treatment plan and the resulting radiotherapy dose distribution. Survival analysis was then performed to investigate potentially statistically significant (*p* > 0.05) correlations between blood dose estimates and LRC, DMFS, and OS, and the relevance of different blood compartments was investigated via the correlation between the dose to the blood compartment in question and the estimated dose to the total circulating blood pool.

### 2.1 Patient datasets and pre‐processing

This study utilized three different datasets: a HNSCC dataset containing 215 patients who had received curative‐intent radiotherapy, a GBM dataset containing a further 180 patients, and a NSCLC dataset containing 490 patients who had received high‐ or standard‐dose radiation therapy and chemotherapy with or without Cetuximab.^29^, ^30^, ^31^, ^32^, ^33^, ^34^, ^35^, ^36^ The aforementioned datasets were chosen because they constituted all publicly available datasets in the cancer imaging archive which provided all information required for inclusion in this study (especially clinical outcome data and relevant timepoints) and can be found there under the identifiers “HNSCC”, “Burdenko‐GBM‐Progression”, and “NSCLC‐Cetuximab”, respectively. There was no patient overlap between the head‐and‐neck cancer dataset used in the prior study and the HNSCC dataset used herein, since for the former, correlations between blood dose estimates and clinical outcomes had already been reported previously.^27^ For every patient, two different types of data were required:

1. **Radiotherapy treatment data,** including the patient's treatment planning CT scan, any structures manually delineated for treatment planning, the patient's clinical radiotherapy treatment plan, and the resulting dose distribution
2. **Patient data,** including the occurrence and time point of at least one clinical outcome as well as characteristics such as sex, age, and staging

Such data was provided for almost all patients in all datasets, but patients for whom any of the files required for the blood dose simulations performed or information regarding the evaluated endpoints were missing had to be excluded during data acquisition, processing, and analysis. No other exclusion criteria were applied. For all three datasets, the distribution of relevant patient characteristics for all patients included in this study is shown in Table 1.

**TABLE 1:** Patient characteristics and the corresponding number and percentage of patients (for categorical variables) or mean ± standard deviation (for continuous variables) for the evaluated patients from the head‐and‐neck squamous cell carcinoma (HNSCC), glioblastoma (GBM), and non‐small cell lung cancer (NSCLC) dataset.

	# of patients (percentage) or Mean ± standard deviation		
Characteristics	HNSCC	GBM	NSCLC
Sex			
Male	168 (84.0%)	95 (53.4%)	258 (59.4%)
Female	32 (16.0%)	81 (45.5%)	176 (40.6%)
Unknown	0 (0.0%)	2 (1.1%)	0 (0.0%)
Age [years]	57.4 ± 9.7	55.3 ± 12.8	63.3 ± 9.0
Smoking history			
Never	73 (36.5%)	–	28 (6.5%)
(Former) Light	22 (11.0%)	–	39 (9.0%)
(Former) Heavy	105 (52.5%)	–	147 (33.9%)
Current	–	–	200 (46.1%)
Unknown	0 (0.0%)	–	20 (4.6%)
T‐stage			
0	6 (3.0%)	–	–
1	35 (17.5%)	–	–
2	62 (31.0%))	–	–
3	58 (29.0%)	–	–
4	39 (19.5%)	–	–
N‐stage			
0	23 (11.5%)	–	–
1	21 (10.5%)	–	–
2	139 (69.5%))	–	–
3	17 (8.5%)	–	–
TNM group stage			
1	3 (1.5%)	–	0 (0.0%)
2	5 (2.5%)	–	0 (0.0%)
3	28 (14.0%)	–	434 (100.0%)
4	164 (82.0%)	–	0 (0.0%)
HPV status			
–	13 (6.5%)	–	–
+	27 (13.5%)	–	–
Unknown	160 (80.0%)	–	–
IDH1/2			
–	–	99 (55.6%)	–
+	–	13 (7.3%)	–
Unknown	–	66 (37.1%)	–
MGMT			
–	–	57 (32.0%)	–
+	–	36 (20.2%)	–
Unknown	–	85 (47.8%)	–
Prescription dose [Gy]	68.7 ± 2.7	59.8 ± 8.8	66.1 ± 6.9
Chemotherapy			
No	80 (40.0%)	–	0 (0.0%)
Yes	120 (60.0%)	–	434 (100.0%)
Surgery			
No	140 (70.0%)	0 (0.0%)	434 (100.0%)
Yes	60 (30.0%)	178 (100.0%)	0 (0.0%)
Locoregional recurrence			
No	153 (76.5%)	–	273 (62.9%)
Yes	47 (23.5%)	–	161 (37.1%)
Distant metastases			
No	175 (87.5%)	–	216 (49.8%)
Yes	25 (12.5%)	–	218 (50.2%)
Death			
No	141 (70.5%)	102 (57.3%)	170 (39.2%)
Yes	59 (29.5%)	76 (42.7%)	264 (60.8%)

**FIGURE 1:**
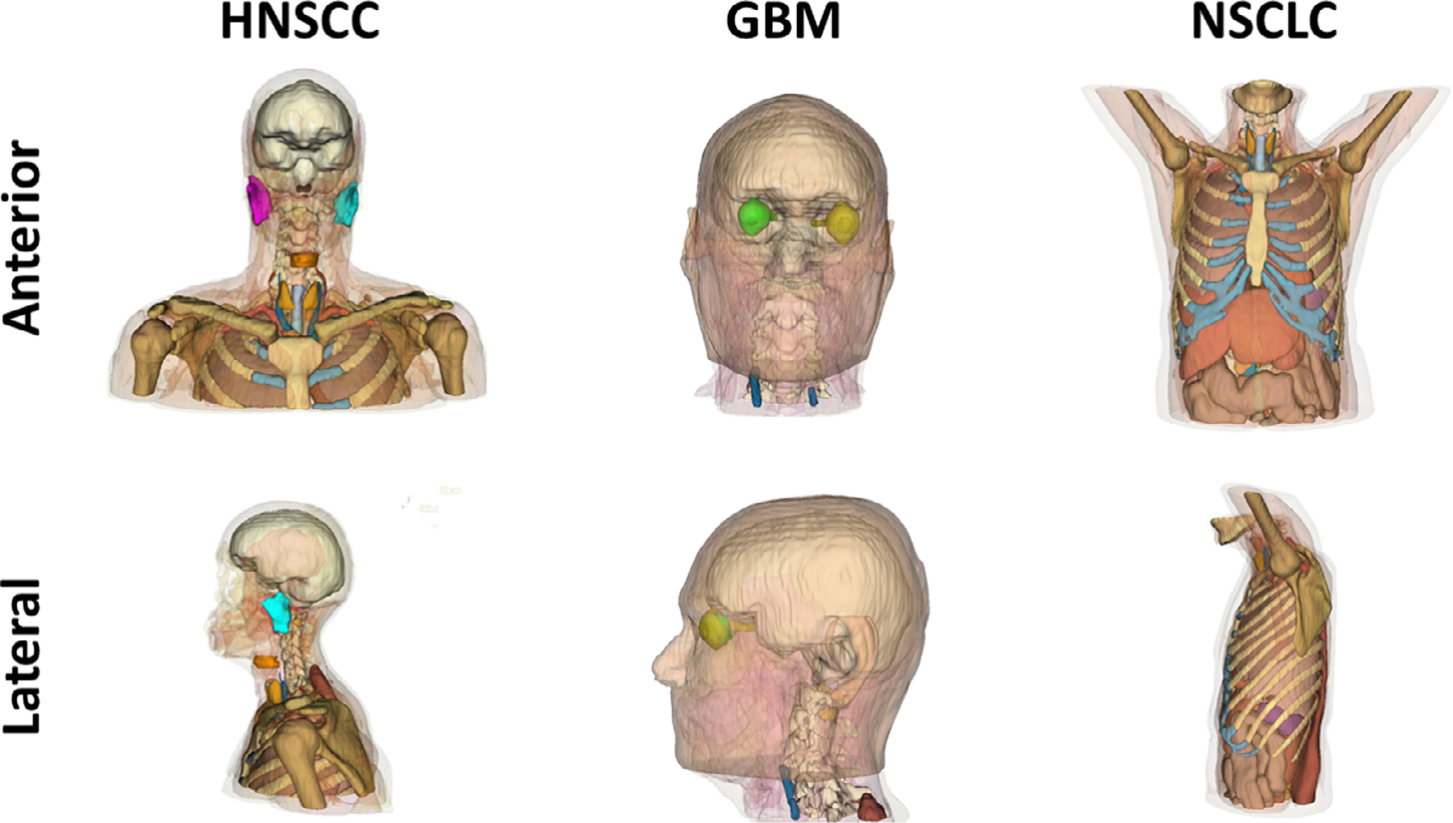
An example of the delineated structures for a patient with (left) head‐and‐neck squamous cell carcinoma (HNSCC), (middle) glioblastoma (GBM), and (right) non‐small cell lung cancer (NSCLC). Figure includes structures which had manually been delineated for treatment planning as well as automatically‐delineated contours. For clarity, the structures depicted do not constitute all delineated structures and are not representative of all structures included in the blood dose models.

Additional organs, blood vessels, and tissues which had not already been manually delineated for radiotherapy treatment planning were automatically delineated on the patient CT scan using TotalSegmentator, an open‐source tool for the automatic delineation of a wide range of contours on CT imaging.^37^ Auto‐contouring included all blood compartments implemented in the HEDOS dynamic blood dose estimation framework as of April 2024 which were not already included in the manually‐delineated patient structures. This generally included organs like the thyroid, a few types of tissue (skeletal muscle and fat) as well as a variety of different blood vessels, including the brachiocephalic veins, internal and common carotid arteries, internal jugular veins, and subclavian arteries. Examples of expanded structure sets for each of the three different patient datasets are shown in Figure 1.

### 2.2 HEDOS blood dose estimation workflow

HEDOS utilizes two independent dynamic models to estimate the radiation dose delivered to circulating blood during radiotherapy: one model which describes the spatial distribution of blood particles in different compartments (organs, blood vessels, and tissues) over time, and one model which concerns the radiation dose delivery. The simulated number of blood particles remained at its default value of 100 000, and total blood volume and cardiac output values were 3.9 and 5.9 L/min for female patients and 5.3 and 6.5 L/min for male patients, based on International Commission on Radiological Protection (ICRP) Report 89.^38^ For the treatment delivery model, patient‐specific parameters such as the number of treatment fractions were read out from a patient's radiotherapy treatment plan, and beam‐on times were calculated based on the assumption of a clinically realistic dose rate of 3 Gy/min.^39^, ^40^ As the beam‐off time between treatment fields has previously been noted to have a negligible impact on results during a prior sensitivity analysis, the beam‐off time was left at its initial value of 10 s.^27^

For blood compartments such as skeletal muscle and fat which were not entirely contained within the patient CT scan, correction factors provided in the literature were applied, under the assumption that any part of the compartment which was not contained within the patient CT was far from the irradiated target(s) and therefore received no radiation dose.^23^ In cases in which a surgical cavity was irradiated (i.e. especially in the GBM dataset), the cavity was excluded from the organ in question for construction of the relevant blood dose model.

HEDOS simulations yield the distribution of doses delivered to all simulated blood particles. To summarize the HEDOS findings, the aforementioned distribution was condensed into three different kinds of metrics: the mean dose over all blood particles (*D*_mean_), the minimum dose delivered to the top X% of blood particles (*D*_X%_), and the percentage of blood particles receiving a dose of at least Y Gy (V_Y Gy_).

In addition to the dynamic HEDOS model, three simplified static blood dose models were constructed to determine whether such simplified approaches exhibited the same potential correlations with adverse clinical outcomes as the dynamic blood dose model. The first static blood dose model simply consisted of the integral body dose (*D*_static,body_) while the second static model consisted of the mean dose within the union of all structures included in the HEDOS model (*D*_static,HEDOS_). Since radiotherapy structure sets were handled in the form of binary label maps in the data evaluation code, addition of the label maps of all structures included in the HEDOS model and evaluation of the dose distribution (e.g. in form of the mean dose) within the structure defined by the resulting label map was straightforward. The effective dose to immune cells (EDIC) framework—a static model which estimates the dose to circulating immune cells based on the blood volume within the lungs, heart, liver, and the rest of the body—could not be applied to the GBM or HNSCC datasets because most of the relevant organs were outside of the field of view of the patient CT scan, but EDIC values were determined and analyzed for the NSCLC dataset.^21^

### 2.3 Data evaluation and statistical analysis

Data analysis was performed in Python. This included plotting of Kaplan–Meier curves for all variables and clinical outcomes considered and log‐rank tests comparing the Kaplan–Meier curves for the 25% of patients with the highest and 25% of patients with the lowest values with respect to the variable in question. Subsequently, univariable Cox proportional hazard modelling was performed for all variables and clinical outcomes considered, and multivariable Cox proportional hazard modelling was likewise performed for all variables and clinical outcomes, without pre‐screening based on univariable analysis results, to avoid exclusion of relevant adjustment variables.^41^ Categorical variables were handled via dummy variables, proportional hazard assumption checks were performed, and multivariable Cox proportional hazard models for the different patient datasets included the following covariates:

‐ **HNSCC**: Patient age, T‐stage, N‐stage, TNM group, whether the patient had received surgery, the chemotherapy agent(s) used, the patient's smoking history, radiotherapy prescription dose, and dynamic blood dose (e.g. *D*_90%_)
‐ **GBM**: Patient age, IDH1/2, MGMT, radiotherapy prescription dose, and dynamic blood dose (e.g. *D*_90%_)
‐ **NSCLC**: Patient age, study arm (high or standard dose radiotherapy with or without the inclusion of Cetuximab), whether radiotherapy was terminated, smoking history, histology, and dynamic blood dose (e.g. *D*_90%_)

To help identify particularly relevant blood compartments, the Pearson correlation coefficient between the dose to the compartment in question and the estimated dose to circulating blood was analyzed.

## 3 RESULTS

The multivariable Cox regression analysis results for the HNSCC dataset are shown in Table 2. During multivariable analysis, T‐stage, smoking history, and dynamic blood dose (in the form of *D*_90%_) exhibited statistically significant (*p* < 0.05) correlations with at least one of the clinical outcomes studied. *D*_90%_, which is presented herein due to the comparatively high radiosensitivity of lymphocytes, exhibited a statistically significant correlation with all three clinical outcomes, with hazard ratios (respective to a change of 1 Gy in the blood dose metric) ranging from 5.4 to 10.9.^42^ The corresponding data for *D*_static,body_ and *D*_static,HEDOS_, which did not exhibit a statistically significant correlation with any clinical outcomes, can be found in Tables S1 and S2, respectively. Although this work largely focussed on *D*_90%_ due to the reported high radiosensitivity of lymphocytes, the data for *D*_mean_ and *D*_10%_ from the dynamic blood dose model, which likewise exhibited a statistically significant correlation with all three clinical outcomes, are provided in Tables S3 and S4.

**TABLE 2:** Multivariable Cox regression analysis results for a variety of patient characteristics and the dynamic blood dose model (*D*_90%_) for the head‐and‐neck squamous cell carcinoma (HNSCC) dataset. Clinical outcomes considered included locoregional control (LRC), distant‐metastasis‐free survival (DMFS), and overall survival (OS). Hazard ratios (HRs) and 95% confidence intervals (95% CI) are only given in cases in which *p* < 0.05.

	LRC		DMFS		OS	
Variable	HR (95% CI)	*p*	HR (95% CI)	*p*	HR (95% CI)	*p*
Age	–	0.798	–	0.583	–	0.449
T‐stage	1.712 (1.133, 2.588)	0.011	–	0.136	1.639 (1.158, 2.320)	0.005
N‐stage	–	0.493	–	0.095	–	0.277
TNM group	–	0.052	–	0.995	–	0.063
Surgery	–	0.247	–	0.610	–	0.252
Chemotherapy agent(s)	–	0.494	–	0.480	–	0.626
Smoking history	1.688 (1.071, 2.662)	0.024	–	0.638	–	0.239
Prescription dose	–	0.350	–	0.728	–	0.362
*D*_90%_	10.899 (2.309, 51.441)	0.003	8.481 (1.060, 67.869)	0.044	5.429 (1.329, 22.174)	0.018

Multivariable Cox regression analysis results for the GBM dataset are shown in Table 3. Only radiotherapy prescription dose exhibited a statistically significant correlation with OS, which was the only clinical outcome information available for this dataset. The corresponding data for *D*_static,body_, *D*_static,HEDOS_, *D*_mean_, and *D*_10%_ can be found in Tables S5–S8.

**TABLE 3:** Multivariable Cox regression analysis results for a variety of patient characteristics and the dynamic blood dose model (*D*_90%_) for the glioblastoma (GBM) dataset. For this dataset, clinical outcome data provided only included overall survival (OS). Hazard ratios (HRs) and 95% confidence intervals (95% CI) are only given in cases in which *p* < 0.05.

	OS	
Variable	HR (95% CI)	*p*
Age	–	0.061
IDH1/2	–	0.752
MGMT	–	0.155
Prescription Dose	0.949 (0.911, 0.988)	0.011
*D*_90%_	–	0.077

Results of the multivariable Cox regression analysis for the NSCLC dataset are shown in Table 4. Age, study arm (high or standard dose radiotherapy with or without the inclusion of Cetuximab), smoking history, and the dynamic blood dose model all exhibited a statistically significant correlation with at least one clinical outcome during multivariable analysis, with a hazard ratio (concerning a change of 1 Gy in the relevant blood dose metric) of approximately 1.2 for *D*_90%_ from the dynamic blood dose model. The corresponding data for the total body dose, the static blood dose model based on the HEDOS blood compartments and EDIC, all of which only exhibited a statistically significant correlation with OS, can be found in Tables S9–11. The corresponding data for *D*_mean_ and *D*_10%_ from the dynamic body dose model, which, consistently with *D*_90%_, exhibited statistically significant correlations with DMFS and OS, can be found in Tables S12–S13.

**TABLE 4:** Multivariable Cox regression analysis results for a variety of patient characteristics and the dynamic blood dose model (*D*_90%_) for the non‐small cell lung cancer (NSCLC) dataset. Clinical outcomes considered included locoregional control (LRC), distant‐metastasis‐free survival (DMFS), and overall survival (OS). Hazard ratios (HRs) and 95% confidence intervals (95% CI) are only given in cases in which *p* < 0.05. Study arm refers to the four study arms of high or standard dose radiotherapy with or without the inclusion of Cetuximab.

	LRC		DMFS		OS	
Variable	HR (95% CI)	*p*	HR (95% CI)	*p*	HR (95% CI)	*p*
Age	–	0.444	0.982 (0.967, 0.997)	0.022	–	0.088
Study arm	1.207 (1.044, 1.395)	0.011	1.158 (1.024, 1.308)	0.019	–	0.633
RT terminated	–	0.510	–	0.504	–	0.890
Smoking history	–	0.401	–	0.978	1.088 (1.009, 1.174)	0.029
Histology	–	0.315	–	0.075	–	0.968
*D*_90%_	–	0.787	1.163 (1.064, 1.272)	0.001	1.225 (1.131, 1.326)	<0.001

**FIGURE 2:**
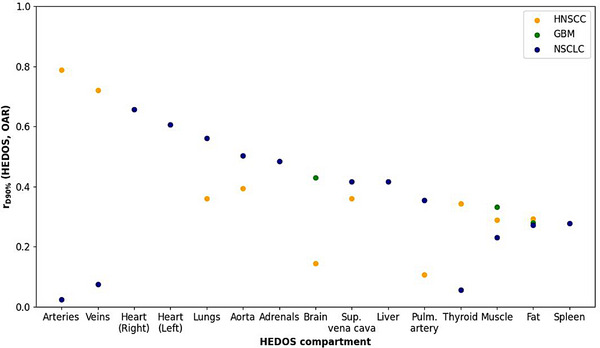
The Pearson correlation coefficient r_D90%_ (HEDOS, OAR) for the correlation between the minimum dose delivered to 90% of the volume of the compartment in question *D*_90%_ from the dynamic blood dose model for all patient datasets and blood compartments. Yellow, green, and blue datapoints depict values for the head‐and‐neck squamous cell carcinoma (HNSCC), glioblastoma (GBM), and non‐small cell lung cancer (NSCLC) dataset, respectively. HEDOS, hematological dose; OAR, organs‐at‐risk.

To identify relevant blood compartments which particularly contributed to the dynamic blood dose model estimates for every treatment site, Pearson correlation coefficients between the minimum dose delivered to 90% of the volume of the compartment in question and *D*_90%_ from the dynamic blood dose model were determined for all datasets and relevant blood compartments and are depicted in Figure 2. Blood compartments with the highest correlation coefficients were large arteries (*r* = 0.789) and veins (*r* = 0.720) for HNSCC, right heart (*r* = 0.657), left heart (*r* = 0.606), and lungs (*r* = 0.561) for NSCLC, and brain (*r* = 0.430) for GBM.

## 4 DISCUSSION

### 4.1 Treatment site‐specific correlations of blood dose estimates with adverse clinical outcomes

The extent of publicly available patient‐specific information differed between datasets, but five patient variables exhibited a statistically significant (*p* < 0.05) correlation with at least one clinical outcome during multivariable analysis: age, T‐stage, smoking history, prescription dose, and study arm (with respect to radiotherapy prescription dose and inclusion of Cetuximab). Although EDIC could not be applied to the HNSCC or GBM datasets because at least one of the relevant OARs was outside of the field‐of‐view of the treatment planning CT scan, any of the static blood dose models only exhibited a statistically significant correlation with a clinical outcome in two out of seven cases studied. The dynamic blood dose metric *D*_90%_, on the other hand, exhibited a statistically significant correlation in five out of seven cases. This indicates that incorporating temporal parameters, such as treatment delivery time and blood flow dynamics, into blood dose frameworks like HEDOS aids in establishing a correlation with adverse clinical outcomes that is not observed for solely static blood dose estimation workflows, even if such a static model only considers the dose deposited in exactly the same structures as are also included in the HEDOS simulations. The importance of considering temporal parameters indicates that it is not solely the cumulative dose delivered to circulating blood but also the way in which it is distributed over individual blood particles that is of importance. Delivering the same dose to a given blood‐rich organ at two different dose rates would result in the same contribution toward static blood dose metrics such as the cumulative body dose or EDIC, but would result in different HEDOS blood dose distributions (and therefore different values for metrics including *D*_90%_). Especially considering the comparatively high radiosensitivity of circulating lymphocytes, such different blood dose distributions would be expected to also result in different patterns of lymphocyte killing, partially explaining the importance of including temporal considerations such as treatment delivery and blood flow dynamics.^42^

The findings of this study are to be considered in the context of various studies which have reported a correlation between blood dose estimates or lymphopenia and adverse clinical outcomes for both head‐and‐neck cancer and NSCLC.^27^, ^43^, ^44^, ^45^, ^46^ Hazard ratios for HNSCC were comparable to those reported for a different head‐and‐neck cancer dataset and higher than those for NSCLC, although this is to be viewed in light of systematically higher *D*_90%_ blood dose estimates in the case of the latter dataset, since hazard ratios for both datasets concerned the risk per Gy of difference in the simulated blood dose metric in question.^27^ Of further consideration are concerns related to multiple testing, since raw p‐values as presented herein are prone to false positives.^47^, ^48^, ^49^ P‐values can instead be corrected with a focus on the family‐wise error rate (i.e., the probability of at least one false positive), for example via the (conservative) Bonferroni or Holm corrections, or with an emphasis on the false discovery rate (i.e., the fraction of false positives), for example via the Benjamini–Hochberg correction. Both approaches come at the cost of false negatives, but e.g., Bonferroni‐corrected *p*‐values can straightforwardly be derived from the number of tests and unadjusted *p* values found in the tables provided in Section 3. Findings which remain statistically significant (*p* < 0.05) even after Bonferroni correction are indicated by particularly low (pre‐adjustment) *p* values and include all reported correlations between dynamic blood dose estimates (in the form of D90%) and DMFS and OS for NSCLC and LRC for HNSCC.

In the GBM dataset, no statistically significant correlation was observed between *D*_static_HEDOS_ or *D*_90%_ from the dynamic blood dose model and OS. In contrast, *D*_static,body_ as well as *D*_mean_ and *D*_10%_ from the dynamic blood dose model did exhibit a statistically significant correlation with OS. It is, however, to be emphasized that the elevated hazard ratios and wide confidence intervals for the latter two metrics suggest that the number of events per predictor/variable was insufficient for the multivariable analysis to yield reliable results.^50^ This was likely due to comparatively short survival times as well as the GBM dataset being the smallest among those included in this study, compounded by the lack of IDH1/2 and/or MGMT data in a considerable number of cases, which further reduced the number of patients available for multivariable analysis. Additional considerations include a lack of some patient and treatment parameters such as baseline lymphocyte counts, which were included in studies which reported statistically significant correlations between lymphopenia and overall survival in other GBM patients.^3^, ^51^, ^52^ Especially for the GBM dataset, for which only a few blood compartments were in sufficient proximity to the irradiated target to be considered relevant, reliance on a simplified static blood metric such as the integral body dose may also simply be sufficient.

For all three patient datasets, a variety of potential cofounding variables was included during multivariable Cox regression analysis, including patient age, radiotherapy prescription dose, smoking history, and the inclusion of additional treatments like surgery or chemotherapy (both for HNSCC and NSCLC), staging (for HNSCC), histology (for NSCLC), and IDH1/2 and MGMT status (for GBM). Even so, the lack of a few potentially relevant patient parameters such as baseline lymphocyte counts which could therefore not be included during multivariable Cox regression analysis affected all three datasets and could partially explain the lack of a correlation between dynamic blood dose estimates and LRC in the NSCLC dataset.^4^, ^28^, ^53^, ^54^, ^55^ Other patient variables such as HPV status were provided for some but not all patients. Information on all three clinical outcomes studied was provided for the HNSCC and NSCLC datasets but not for the GBM patients. A potential correlation between evaluated patient and blood dose metrics and LRC and/or DMFS in the latter dataset could therefore not be evaluated.

### 4.2 Assumptions of the current workflow

The current version of the dynamic blood dose model is limited to circulating lymphocytes, which constitute only approximately 2% of the total lymphocyte pool.^56^, ^57^ Although the first model considering lymphoid organs in addition to the bloodstream has recently been reported, irradiation of circulating blood has also been suggested to be crucial because radiation‐induced lymphopenia has been reported in treatment sites in which little lymphoid tissue is irradiated.^13^^,^ ^52^, ^58^, ^59^ Although lymph nodes play an important role as lymphocyte repositories and their irradiation is therefore very relevant to lymphocyte depletion, a lack of a reliable segmentation method necessitated the omission of the lymph nodes as a separate compartment.^65^, ^66^, ^67^, ^68^, ^69^, ^70^, ^71^ The dose delivered to the lymph nodes was, however, considered as part of other more general healthy tissue blood compartments, as was the radiation dose delivered to bone marrow. The impact of especially the latter approach is expected to be very limited, since the mean dose within all delineated bones was on the order of only a fraction of a gray per treatment fraction.^38^ Further random errors in simulated blood dose values resulted from changes in patient anatomy (which may be considerable in head‐and‐neck and lung cancer patients) as well as uncertainties in the delineated structures, which likewise affect dose distributions determined for clinical radiotherapy treatment planning.^37^, ^60^, ^61^, ^62^, ^63^, ^64^ Because of the considerable size of the patient dataset and the high (> 0.9) Dice coefficients which have previously been reported to be associated with the auto‐contouring model used, auto‐delineated structures did not undergo manual corrections, contributing to blood dose model uncertainties, especially for difficult‐to‐delineate organs and blood vessels in close proximity to the irradiated target(s).^37^

A clinically realistic value of 3 Gy/min was applied in all dynamic blood dose simulations. However, maximum dose rates over the course of treatment delivery may vary by several Gy/min, and considerable portions of even the target volume may receive dose rates < 1 Gy/min.^72^, ^73^ It has previously been shown that such reductions in dose rate increase the number of blood particles receiving low doses while simultaneously reducing the dose delivered to blood particles receiving particularly high doses.^23^ Although the magnitude of this effect is expected to be treatment site‐ and even patient‐specific, for a sample patient with hepatocellular carcinoma, even a 30‐fold dose rate reduction resulting in a delivery time increase from 10 to 300 s only increased *D*_90%_ from 11.9 to 15.3 Gy.^26^ The impact of clinically realistic deviations from the utilized dose rate of 3 Gy/min is therefore expected to be limited.

Lastly, it is to be emphasized that the blood dose estimates determined for this work are theoretical, since experimental determination of the dose‐volume histogram of the circulating blood is infeasible. A similar application of the dynamic blood dose model HEDOS has, however, shown a statistically significant decrease in post‐radiotherapy absolute lymphocyte counts at higher simulated doses to circulating blood.^26^ Although it is not possible to experimentally verify the doses delivered to the circulating blood particles for the patients in question, blood dose estimates from the dynamic blood dose simulations performed therefore still appear to reflect differences in blood dose acquisition patterns between different patients, resulting in increased killing of circulating lymphocytes for patients with higher simulated blood dose estimates.

### 4.3 Identification of relevant blood dose metrics and blood compartments

For the HNSCC and NSCLC datasets, all metrics extracted from the dynamic blood dose model (*D*_90%_, *D*_mean_, and *D*_10%_), exhibited statistically significant correlations with the same clinical outcomes, indicating the importance of the estimated dose delivered to circulating blood in general rather than a clear preference for a specific dynamic blood dose metric in particular. As reflected in Figure 2, in terms of relevant blood compartments, similar correlation coefficients were found regarding the compartment dose and the dynamic blood dose estimate for general healthy tissue compartments such as fat and skeletal muscle for all three investigated treatment sites.

For the HNSCC dataset, large arteries and veins exhibited the highest correlation coefficients, indicating that their inclusion during radiotherapy treatment planning may particularly benefit dynamic blood dose estimates. Since such blood vessels are currently not generally considered during radiotherapy treatment planning, their delineation and inclusion during treatment plan optimization may constitute a comparatively straightforward way to reduce the blood dose, as it may be feasible to redistribute some of the dose delivered to the aforementioned blood vessels to nearby general healthy tissues instead.

Likely due to most delineated arteries and vessels having been in the head‐and‐neck region and therefore receiving little dose in the NSCLC patient cases, the same trend was not observed in this patient dataset, with the heart and lungs being associated with the highest correlation coefficients instead. In such cases, blood dose reductions may therefore instead be achievable via e.g. higher weighting/prioritization of the aforementioned organs during treatment plan optimization or the selection of beam angles which may be able to reduce the radiation dose delivered to the specific OARs in question. Within the GBM patient dataset, the healthy part of the brain exhibited a higher correlation coefficient than other general healthy tissue compartments.

Although the inclusion of the dose delivered to circulating blood during radiotherapy treatment planning may at times be achievable by redistributing the relevant dose to nearby general healthy tissue, a trade‐off between the dose delivered to circulating blood and target coverage and/or the sparing of other OARs is to be expected in some cases. In such situations, planning objective weights for the structures in question and/or the chosen beam geometry can be adjusted until an acceptable balance between the different considerations is found.

## 5 CONCLUSIONS

HEDOS blood dose estimates exhibited a statistically significant (*p* < 0.05) correlation with adverse clinical outcomes in five out of seven cases, whereas static blood dose models only exhibited such correlations in two cases. Considerations of temporal aspects such as treatment delivery time and blood flow dynamics were therefore essential to the observation of some of the reported correlations with adverse clinical outcomes. The investigated metrics extracted from the dynamic blood dose model generally exhibited statistically significant correlations with the same clinical outcomes, indicating the importance of the dose delivered to the circulating blood in general rather than a clear preference for one dynamic blood dose metric in particular. In the HNSCC, GBM, and NSCLC datasets, particularly relevant blood compartments were determined to be large arteries and veins, the healthy portion of the brain, and the heart and lungs, respectively. Future work will focus on including the blood compartment identified as most relevant herein during radiotherapy treatment planning to reduce the radiation dose delivered to circulating blood. Such blood dose optimization strategies may be especially of interest for NSCLC patients receiving immunotherapy as well as radiation, for whom severe RIL has been shown to (partially) compromise the clinical benefits of immunotherapy. For such patient populations, studies focusing on post‐radiotherapy lymphocyte counts as well as early clinical outcomes could be performed to establish whether blood dose optimization approaches may (partially) counteract the aforementioned negative effects of radiation delivery on the benefits of immunotherapy treatments.^74^, ^75^

## AUTHOR CONTRIBUTIONS

**Sebastian Tattenberg**: Conceptualization; data collection; methodology contribution; manuscript preparation. **Jungwook Shin**: Methodology contribution; manuscript contribution. **Cornelia Hoehr**: Methodology contribution; manuscript contribution. **Xuanfeng Ding**: Methodology contribution; manuscript contribution. **Rohan L. Deraniyagala**: Methodology contribution; manuscript contribution. **Wonmo Sung**: Conceptualization; methodology contribution; manuscript contribution.

## CONFLICT OF INTEREST STATEMENT

Xuanfeng Ding received honorariums from IBA and Elekta's speaker bureau outside the work presented here. Xuanfeng Ding received industrial research funding from IBA and Elekta outside this work.

## Supporting information




^Supporting Information^
