# Supplementary material for: Dynamic blood dose estimates in radiotherapy and correlations with adverse clinical outcomes in brain, head‐and‐neck, and lung cancer patients
Source: J Appl Clin Med Phys. 2025 Dec 29;27(1):e70341. doi: 10.1002/acm2.70341 (PMC12745927; doi:10.1002/acm2.70341)
Supplement: Supplementary file 1 — Supporting Information [file ACM2-27-e70341-s001.docx]

**Supplementary documents**

Table S1 Multivariable Cox regression analysis results for a variety of patient characteristics and the total body dose (D_static,body_) for the head-and-neck squamous cell carcinoma (HNSCC) dataset. Clinical outcomes considered included locoregional control (LRC), distant-metastasis-free survival (DMFS), and overall survival (OS). Hazard ratios (HRs) and 95% confidence intervals (95% CI) are only given in cases in which p < 0.05.

| **Variable** | **LRC** | | **DMFS** | | **OS** | |
| --- | --- | --- | --- | --- | --- | --- |
|  | **HR**  **(95% CI)** | **p** | **HR**  **(95% CI)** | **p** | **HR**  **(95% CI)** | **p** |
| Age | - | 0.359 | - | 0.301 | - | 0.722 |
| T-stage | 1.751 (1.162, 2.64) | 0.007 | - | 0.058 | 1.623 (1.150, 2.291) | 0.006 |
| N-stage | - | 0.434 | 3.392 (1.159, 9.927) | 0.026 | - | 0.334 |
| TNM Group | - | 0.112 | - | 0.995 | - | 0.092 |
| Surgery | - | 0.222 | - | 0.860 | - | 0.172 |
| Chemotherapy Agent(s) | - | 0.345 | - | 0.672 | - | 0.848 |
| Smoking History | 1.614 (1.051, 2.481) | 0.029 | - | 0.782 | - | 0.337 |
| Prescription Dose | - | 0.222 | - | 0.868 | - | 0.280 |
| D_static,body_ | - | 0.838 | - | 0.159 | - | 0.429 |

Table S2 Multivariable Cox regression analysis results for a variety of patient characteristics and the static blood dose model based on the HEDOS blood compartments (D_static,HEDOS_) for the head-and-neck squamous cell carcinoma (HNSCC) dataset. Clinical outcomes considered included locoregional control (LRC), distant-metastasis-free survival (DMFS), and overall survival (OS). Hazard ratios (HRs) and 95% confidence intervals (95% CI) are only given in cases in which p < 0.05.

| **Variable** | **LRC** | | **DMFS** | | **OS** | |
| --- | --- | --- | --- | --- | --- | --- |
|  | **HR**  **(95% CI)** | **p** | **HR**  **(95% CI)** | **p** | **HR**  **(95% CI)** | **p** |
| Age | - | 0.349 | - | 0.209 | - | 0.957 |
| T-stage | 1.775 (1.173, 2.687) | 0.007 | - | 0.083 | 1.656 (1.172, 2.341) | 0.004 |
| N-stage | - | 0.446 | - | 0.088 | - | 0.240 |
| TNM Group | - | 0.109 | - | 0.995 | - | 0.092 |
| Surgery | - | 0.229 | - | 0.755 | - | 0.285 |
| Chemotherapy Agent(s) | - | 0.494 | - | 0.393 | - | 0.604 |
| Smoking History | 1.646 (1.048, 2.586) | 0.030 | - | 0.632 | - | 0.237 |
| Prescription Dose | - | 0.274 | - | 0.771 | - | 0.288 |
| D_static.HEDOS_ | - | 0.721 | - | 0.114 | - | 0.561 |

Table S3 Multivariable Cox regression analysis results for a variety of patient characteristics and the dynamic blood dose model (D_mean_) for the head-and-neck squamous cell carcinoma (HNSCC) dataset. Clinical outcomes considered included locoregional control (LRC), distant-metastasis-free survival (DMFS), and overall survival (OS). Hazard ratios (HRs) and 95% confidence intervals (95% CI) are only given in cases in which p < 0.05.

| **Variable** | **LRC** | | **DMFS** | | **OS** | |
| --- | --- | --- | --- | --- | --- | --- |
|  | **HR**  **(95% CI)** | **p** | **HR**  **(95% CI)** | **p** | **HR**  **(95% CI)** | **p** |
| Age | - | 0.801 | - | 0.609 | - | 0.464 |
| T-stage | 1.694 (1.123, 2.556) | 0.012 | - | 0.156 | 1.620 (1.146, 2.290) | 0.006 |
| N-stage | - | 0.494 | - | 0.096 | - | 0.286 |
| TNM Group | - | 0.053 | - | 0.995 | - | 0.068 |
| Surgery | - | 0.250 | - | 0.596 | - | 0.255 |
| Chemotherapy Agent(s) | - | 0.497 | - | 0.471 | - | 0.619 |
| Smoking History | 1.698 (1.075, 2.681) | 0.023 | - | 0.633 | - | 0.234 |
| Prescription Dose | - | 0.366 | - | 0.773 | - | 0.373 |
| D_mean_ | 9.255 (2.255, 37.992) | 0.002 | 8.213 (1.299, 51.935) | 0.025 | 4.576 (1.28, 16.359) | 0.019 |

Table S4 Multivariable Cox regression analysis results for a variety of patient characteristics and the dynamic blood dose model (D_10%_) for the head-and-neck squamous cell carcinoma (HNSCC) dataset. Clinical outcomes considered included locoregional control (LRC), distant-metastasis-free survival (DMFS), and overall survival (OS). Hazard ratios (HRs) and 95% confidence intervals (95% CI) are only given in cases in which p < 0.05.

| **Variable** | **LRC** | | **DMFS** | | **OS** | |
| --- | --- | --- | --- | --- | --- | --- |
|  | **HR**  **(95% CI)** | **p** | **HR**  **(95% CI)** | **p** | **HR**  **(95% CI)** | **p** |
| Age | - | 0.813 | - | 0.621 | - | 0.483 |
| T-stage | 1.677 (1.113, 2.527) | 0.013 | - | 0.179 | 1.604 (1.135, 2.265) | 0.007 |
| N-stage | - | 0.497 | - | 0.098 | - | 0.293 |
| TNM Group | - | 0.054 | - | 0.995 | - | 0.072 |
| Surgery | - | 0.252 | - | 0.584 | - | 0.258 |
| Chemotherapy Agent(s) | - | 0.500 | - | 0.460 | - | 0.614 |
| Smoking History | 1.707 (1.08,2.699) | 0.022 | - | 0.627 | - | 0.229 |
| Prescription Dose | - | 0.380 | - | 0.816 | - | 0.382 |
| D_10%_ | 7.847 (2.177, 28.291) | 0.002 | 7.579 (1.476, 38.918) | 0.015 | 3.921 (1.235, 12.444) | 0.020 |

Table S5 Multivariable Cox regression analysis results for a variety of patient characteristics and the total body dose (D_static,body_) for the glioblastoma (GBM) dataset. For this dataset, clinical outcome data provided only included overall survival (OS). Hazard ratios (HRs) and 95% confidence intervals (95% CI) are only given in cases in which p < 0.05.

| **Variable** | **OS** | |
| --- | --- | --- |
|  | **HR**  **(95% CI)** | **p** |
| Age | 1.043 (1.004, 1.083) | 0.031 |
| IDH1/2 | - | 0.940 |
| MGMT | - | 0.595 |
| Prescription Dose | 0.964 (0.930, 0.999) | 0.046 |
| D_static,body_ | 1.000 (1.000, 1.000) | 0.020 |

Table S6 Multivariable Cox regression analysis results for a variety of patient characteristics and the static blood dose model based on the HEDOS blood compartments (D_static,HEDOS_) for the glioblastoma (GBM) dataset. For this dataset, clinical outcome data provided only included overall survival (OS). Hazard ratios (HRs) and 95% confidence intervals (95% CI) are only given in cases in which p < 0.05.

| **Variable** | **OS** | |
| --- | --- | --- |
|  | **HR**  **(95% CI)** | **p** |
| Age | 1.040 (1.003, 1.078) | 0.033 |
| IDH1/2 | - | 0.681 |
| MGMT | - | 0.246 |
| Prescription Dose | 0.955 (0.920, 0.993) | 0.019 |
| D_static,HEDOS_ | - | 0.114 |

Table S7 Multivariable Cox regression analysis results for a variety of patient characteristics and the dynamic blood dose model (D_mean_) for the glioblastoma (GBM) dataset. For this dataset, clinical outcome data provided only included overall survival (OS). Hazard ratios (HRs) and 95% confidence intervals (95% CI) are only given in cases in which p < 0.05.

| **Variable** | **OS** | |
| --- | --- | --- |
|  | **HR**  **(95% CI)** | **p** |
| Age | 1.037 (1.001, 1.074) | 0.044 |
| IDH1/2 | - | 0.711 |
| MGMT | - | 0.279 |
| Prescription Dose | 0.948 (0.912, 0.986) | 0.008 |
| D_mean_ | 468.473 (1.477, 148583.219) | 0.036 |

Table S8 Multivariable Cox regression analysis results for a variety of patient characteristics and the dynamic blood dose model (D_10%_) for the glioblastoma (GBM) dataset. For this dataset, clinical outcome data provided only included overall survival (OS). Hazard ratios (HRs) and 95% confidence intervals (95% CI) are only given in cases in which p < 0.05.

| **Variable** | **OS** | |
| --- | --- | --- |
|  | **HR**  **(95% CI)** | **p** |
| Age | 1.039 (1.002, 1.076) | 0.036 |
| IDH1/2 | - | 0.695 |
| MGMT | - | 0.366 |
| Prescription Dose | 0.951 (0.915, 0.987) | 0.009 |
| D_10%_ | 80.221 (1.538, 4185.225) | 0.030 |

Table S9 Multivariable Cox regression analysis results for a variety of patient characteristics and the total body dose (D_static,body_) for the non-small cell lung cancer (NSCLC) dataset. Clinical outcomes considered included locoregional control (LRC), distant-metastasis-free survival (DMFS), and overall survival (OS). Hazard ratios (HRs) and 95% confidence intervals (95% CI) are only given in cases in which p < 0.05. Study arm refers to the four study arms of high or standard dose radiotherapy with or without inclusion of Cetuximab.

| **Variable** | **LRC** | | **DMFS** | | **OS** | |
| --- | --- | --- | --- | --- | --- | --- |
|  | **HR**  **(95% CI)** | **p** | **HR**  **(95% CI)** | **p** | **HR**  **(95% CI)** | **p** |
| Age | - | 0.424 | 0.982 (0.967, 0.997) | 0.017 | - | 0.148 |
| Study Arm | 1.203 (1.041, 1.389) | 0.012 | 1.163 (1.029, 1.314) | 0.015 | - | 0.407 |
| RT Terminated | - | 0.472 | - | 0.664 | - | 0.575 |
| Smoking History | - | 0.357 | - | 0.869 | 1.082 (1.003, 1.168) | 0.043 |
| Histology | - | 0.181 | - | 0.113 | - | 0.793 |
| D_static,body_ | - | 0.366 | - | 0.755 | 1.000 (1.000, 1.000) | 0.039 |

Table S10 Multivariable Cox regression analysis results for a variety of patient characteristics and the static blood dose model based on the HEDOS blood compartments (D_static,HEDOS_) for the non-small cell lung cancer (NSCLC) dataset. Clinical outcomes considered included locoregional control (LRC), distant-metastasis-free survival (DMFS), and overall survival (OS). Hazard ratios (HRs) and 95% confidence intervals (95% CI) are only given in cases in which p < 0.05. Study arm refers to the four study arms of high or standard dose radiotherapy with or without inclusion of Cetuximab.

| **Variable** | **LRC** | | **DMFS** | | **OS** | |
| --- | --- | --- | --- | --- | --- | --- |
|  | **HR**  **(95% CI)** | **p** | **HR**  **(95% CI)** | **p** | **HR**  **(95% CI)** | **p** |
| Age | - | 0.434 | 0.983 (0.968, 0.998) | 0.025 | - | 0.109 |
| Study Arm | 1.210 (1.046, 1.399) | 0.010 | 1.166 (1.031, 1.317) | 0.014 | - | 0.517 |
| RT Terminated | - | 0.483 | - | 0.687 | - | 0.729 |
| Smoking History | - | 0.408 | - | 0.885 | 1.089 (1.007, 1.177) | 0.032 |
| Histology | - | 0.307 | - | 0.101 | - | 0.919 |
| D_static,HEDOS_ | - | 0.803 | - | 0.673 | 1.201 (1.067, 1.352) | 0.002 |

Table S11 Multivariable Cox regression analysis results for a variety of patient characteristics and the effective dose to immune cells (EDIC) blood dose estimate for the non-small cell lung cancer (NSCLC) dataset. Clinical outcomes considered included locoregional control (LRC), distant-metastasis-free survival (DMFS), and overall survival (OS). Hazard ratios (HRs) and 95% confidence intervals (95% CI) are only given in cases in which p < 0.05. Study arm refers to the four study arms of high or standard dose radiotherapy with or without inclusion of Cetuximab.

| **Variable** | **LRC** | | **DMFS** | | **OS** | |
| --- | --- | --- | --- | --- | --- | --- |
|  | **HR**  **(95% CI)** | **p** | **HR**  **(95% CI)** | **p** | **HR**  **(95% CI)** | **p** |
| Age | - | 0.430 | 0.982 (0.967, 0.997) | 0.021 | - | 0.131 |
| Study Arm | 1.212 (1.047, 1.402) | 0.010 | 1.157 (1.024, 1.308) | 0.020 | - | 0.682 |
| RT Terminated | - | 0.499 | - | 0.698 | - | 0.500 |
| Smoking History | - | 0.385 | - | 0.764 | - | 0.089 |
| Histology | - | 0.294 | - | 0.075 | - | 0.862 |
| EDIC | - | 0.706 | - | 0.161 | 1.297 (1.124, 1.496) | <0.001 |

Table S12 Multivariable Cox regression analysis results for a variety of patient characteristics and the dynamic blood dose model (D_mean_) for the non-small cell lung cancer (NSCLC) dataset. Clinical outcomes considered included locoregional control (LRC), distant-metastasis-free survival (DMFS), and overall survival (OS). Hazard ratios (HRs) and 95% confidence intervals (95% CI) are only given in cases in which p < 0.05. Study arm refers to the four study arms of high or standard dose radiotherapy with or without inclusion of Cetuximab.

| **Variable** | **LRC** | | **DMFS** | | **OS** | |
| --- | --- | --- | --- | --- | --- | --- |
|  | **HR**  **(95% CI)** | **p** | **HR**  **(95% CI)** | **p** | **HR**  **(95% CI)** | **p** |
| Age | - | 0.444 | 0.982 (0.967, 0.997) | 0.022 | - | 0.088 |
| Study Arm | 1.207 (1.044, 1.395) | 0.011 | 1.160 (1.026, 1.311) | 0.018 | - | 0.607 |
| RT Terminated | - | 0.512 | - | 0.507 | - | 0.884 |
| Smoking History | - | 0.402 | - | 0.961 | 1.087 (1.007, 1.172) | 0.032 |
| Histology | - | 0.315 | - | 0.076 | - | 0.972 |
| D_mean_ | - | 0.770 | 1.145 (1.056, 1.241) | 0.001 | 1.201 (1.117, 1.291) | <0.001 |

Table S13 Multivariable Cox regression analysis results for a variety of patient characteristics and the dynamic blood dose model (D_10%_) for the non-small cell lung cancer (NSCLC) dataset. Clinical outcomes considered included locoregional control (LRC), distant-metastasis-free survival (DMFS), and overall survival (OS). Hazard ratios (HRs) and 95% confidence intervals (95% CI) are only given in cases in which p < 0.05. Study arm refers to the four study arms of high or standard dose radiotherapy with or without inclusion of Cetuximab.

| **Variable** | **LRC** | | **DMFS** | | **OS** | |
| --- | --- | --- | --- | --- | --- | --- |
|  | **HR**  **(95% CI)** | **p** | **HR**  **(95% CI)** | **p** | **HR**  **(95% CI)** | **p** |
| Age | - | 0.444 | 0.982 (0.967, 0.997) | 0.022 | - | 0.088 |
| Study Arm | 1.207 (1.044, 1.395) | 0.011 | 1.161 (1.028, 1.313) | 0.016 | - | 0.584 |
| RT Terminated | - | 0.513 | - | 0.510 | - | 0.880 |
| Smoking History | - | 0.403 | - | 0.947 | 1.085 (1.006, 1.171) | 0.035 |
| Histology | - | 0.316 | - | 0.076 | - | 0.974 |
| D_10%_ | - | 0.754 | 1.130 (1.049, 1.216) | 0.001 | 1.180 (1.104, 1.261) | <0.001 |
